# Scientific facts improve cannabis perception and public opinion: results from Sinaloa, México

**DOI:** 10.1038/s41598-023-44185-5

**Published:** 2023-10-12

**Authors:** Josué Camberos-Barraza, Juan F. Osuna-Ramos, Ángel R. Rábago-Monzón, Luis F. Quiñonez-Angulo, Héctor R. González-Peña, Alan A. Pérez-Ramos, Alejandro Camacho-Zamora, Héctor López-Lazcano, Marco A. Valdez-Flores, Carla E. Angulo-Rojo, Alma M. Guadrón-Llanos, Verónica J. Picos-Cárdenas, Claudia D. Norzagaray-Valenzuela, Alberto K. De la Herrán-Arita

**Affiliations:** 1 Faculty of Medicine, Autonomous University of Sinaloa, Culiacán, Mexico; 2 Faculty of Economic and Social Sciences, Autonomous University of Sinaloa, Culiacán, Mexico; 3 “Sinaloa Verde de a Devis” Collective, Culiacán, Mexico; 4 Faculty of Biology, Autonomous University of Sinaloa, Culiacán, Mexico

**Keywords:** Human behaviour, Drug regulation

## Abstract

Cannabis, the most prevalent drug in Latin America, has long been associated with the state of Sinaloa, Mexico, known for its cultivation and distribution. Despite increasing global acceptance, cannabis use remains stigmatized in Mexican society, driven by perceptions of it as a highly psychoactive and addictive substance lacking medicinal or industrial value. This study investigates the impact of scientific information on societal perceptions of cannabis in Sinaloa. A large convenience sample of 3162 individuals from Sinaloa participated in this research, responding to a questionnaire on cannabis consumption and attitudes. Participants were then subjected to an intervention consisting of an informative briefing based on the documents “Using Evidence to Talk About Cannabis” and “State of the Evidence: cannabis use and regulation" by the International Centre for Science in Drug Policy. After the intervention, participants' attitudes were immediately reevaluated through the same questionnaire, allowing for a comparison of pre- and post-intervention responses. The results indicate that the intervention (providing scientific information) significantly influenced attitudes toward cannabis, with education and age playing prominent roles in its effectiveness. Notably, the intervention fostered more positive or more neutral attitudes, potentially reducing stigma and promoting a better-informed perspective on cannabis. This study highlights the pivotal role of evidence in shaping informed citizens' views, while underscoring the importance of countering misinformation for societal progress. These findings have significant implications for forthcoming cannabis policy modifications in Mexico, emphasizing the necessity of engaging knowledgeable individuals in policy decisions to address the violence and inequalities associated with the illicit drug trade, particularly in Sinaloa.

## Introduction

Cannabis holds the distinction of being the most prevalent illicit substance utilized in Latin America. Recent data has demonstrated a substantial increase in the consumption of recreational drugs, particularly cannabis and cocaine, within Mexico over the past decade^1,2^. However, despite the progressive international acceptance of cannabis, its usage continues to be viewed as deviant within Mexican society^1,2^.

Sinaloa stands as one of the primary global hubs for cannabis production. While cannabis cultivation and smuggling traditionally targeted the United States market, it has expanded to include a domestic market. This shift is evident in the escalating levels of drug-related violence and the strain on local penitentiary systems. The scourge of drug trafficking, accompanied by the consequent violence and corruption, constitutes a significant factor in the social taboo surrounding illegal drugs in Mexico^3,4^.

Nonetheless, the legal status of cannabis in Mexico has undergone notable transformations recently. The approval of the General Health Law in 2017 sanctioned the medicinal use of cannabis, provided there is a medical prescription and a permit from the Federal Commission for the Protection against Sanitary Risks (COFEPRIS). Subsequently, in 2018, the Supreme Court of Justice of the Nation deemed the prohibition of recreational cannabis use in Mexico unconstitutional^5^. However, it is important to note that this ruling does not equate to legalizing recreational cannabis use. It applies solely to the individual who initiated the legal challenge but sets a significant precedent for future cases.

More recently, in 2021, the Senate of the Republic approved the Federal Law for the Regulation of Cannabis, establishing a comprehensive legal framework encompassing recreational, medicinal, and industrial applications of cannabis. However, this law still awaits deliberation and approval by the Chamber of Deputies before it can be enacted^6^. Possessing small quantities of cannabis for personal use is not punishable under Mexican law as long as it does not occur in public spaces or in proximity to minors. Nevertheless, the cultivation and sale of cannabis without the requisite permits remain illegal and subject to criminal penalties.

Despite these advancements, cannabis continues to face societal non-acceptance in Sinaloa, a state that has consistently played a prominent role in producing and distributing illicit drugs.

This situation has resulted in unintended collateral damage, encompassing street violence, occupational hazards faced by law enforcement personnel, governmental corruption, and various social and economic drawbacks. Mexico's approach to combating drug-related issues has led to the loss of numerous lives among law enforcement agents (including police and military personnel), with civilian casualties numbering in the hundreds of thousands.

Regrettably, the prohibitionist approach to drug control, prioritizing criminalization and enforcement, has created a lucrative and clandestine drug trade. This underground market thrives on the high demand for illegal substances, such as cannabis, and is often accompanied by violence and criminal activities. Trafficking organizations vie for control over lucrative drug routes, leading to deadly confrontations among rival groups and with law enforcement (two recent significant occurrences in Sinaloa, commonly referred to as "Black Thursdays", marked pivotal junctures in the region's history). As a result, civilian casualties have escalated, and communities living near drug trafficking routes become inadvertent victims of violence and instability. Moreover, the intertwining of drug trafficking with corruption at various levels of government further exacerbates the crisis. Corrupt officials enable drug cartels by turning a blind eye to their activities, accepting bribes, or actively participating in illicit operations^3,4^. This collusion undermines the rule of law, erodes public trust in institutions, and fosters a sense of impunity among traffickers. The consequences of this corruption are severe, with law enforcement agencies being compromised and unable to effectively combat drug-related violence^4,7^.

This prevailing circumstance has significantly shaped the public perception of cannabis in Mexico, particularly in Sinaloa. However, it is important to note that the association of violence and perversion with the drug trade is not the sole justification for cannabis prohibition.

Within Mexican society, cannabis is commonly perceived as a highly potent and addictive substance devoid of any medicinal or industrial utility. Moreover, cannabis consumption is viewed as a behavior that corrodes the social fabric, leading to the social exclusion of individuals utilizing cannabis for either medical or recreational purposes. Furthermore, cannabis is still regarded as a gateway drug that predisposes individuals to use more potent substances. Unfortunately, these assertions often lack substantial support from the available scientific evidence^4,8,9^.

This lack of awareness among both the general populace and policymakers could significantly influence the formulation of public policies. There is an urgent need for a paradigm shift toward evidence-based policies. Several studies have elucidated various individual factors associated with illicit drug use in Mexico, such as age, gender, and educational attainment, among others^4^. However, the objective of the present study is to ascertain the prevalence and perception of cannabis consumption in the Mexican state of Sinaloa, while also examining the impact of disseminating scientific facts about cannabis on attitudes and public perception.

## Materials and methods

The study was conducted spanning the period from November 2022 to March 2023. The surveyors of this study, last year medical students, received comprehensive training to diligently minimize selection bias, response bias, confirmation bias, and social desirability throughout the process. In addition, the surveyors were trained to avoid biased language and leading questions.

All participants provided informed consent, and the research procedures were approved by the ethics committee of the Faculty of Medicine at the Autonomous University of Sinaloa. All methods were performed in accordance with the relevant guidelines and regulations.

### Recruitment of participants

**Figure 1:**
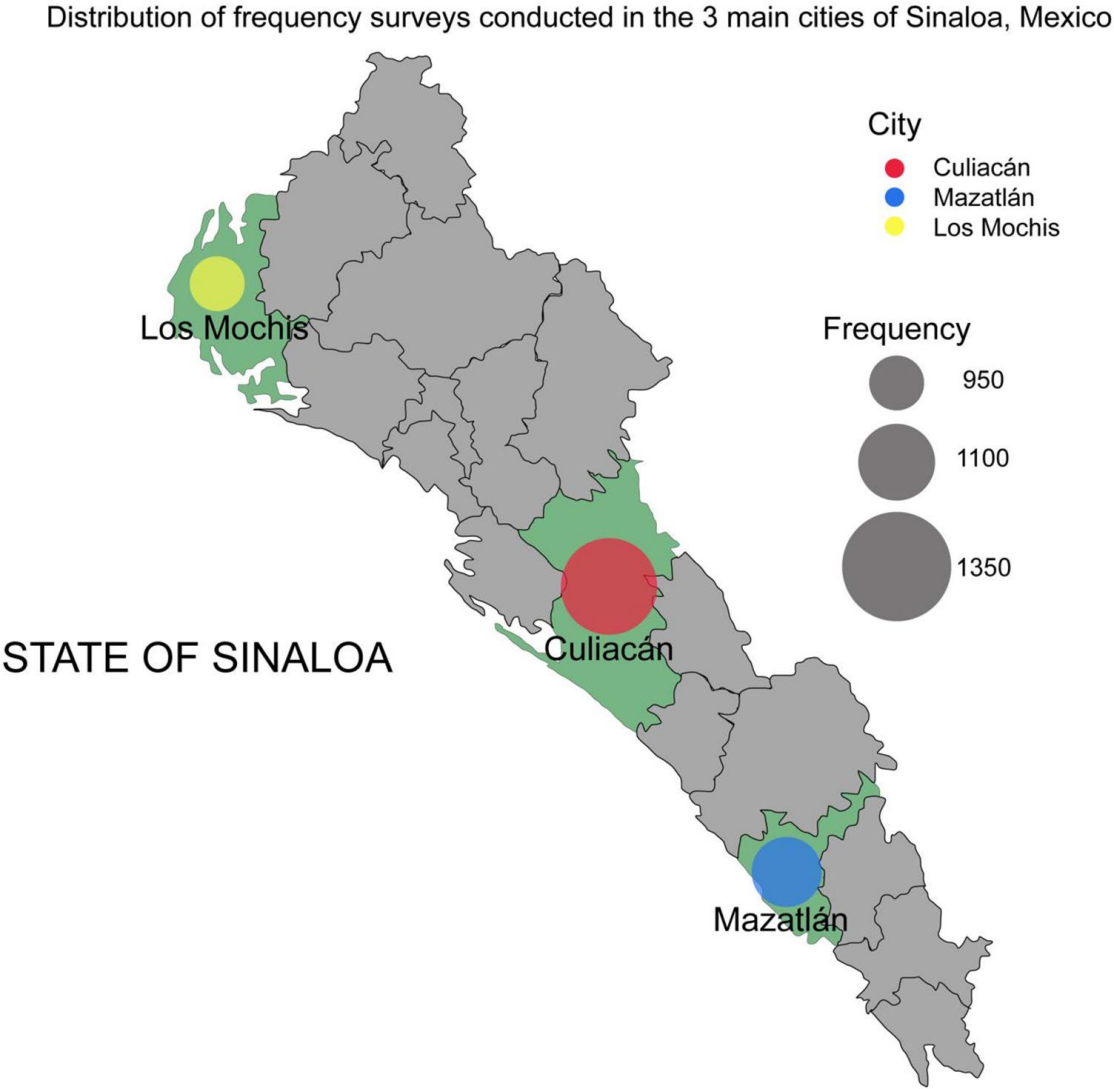
Geographic distribution of survey frequencies across three cities in Sinaloa, Mexico. This scatter plot superimposed on a map depicts the locations and frequencies of surveys conducted in Culiacán (red), Mazatlán (blue), and Los Mochis (yellow). Each point's size represents the relative frequency of surveys conducted in each city. The map background, retrieved from Google Maps, provides a geographical context for the survey locations. This figure was created by AKDA in Adobe Illustrator 24.0 (www.adobe.com/es/products/illustrator.html).

A total of 3162 adults residing in the primary cities of Sinaloa, namely Culiacán (n = 1133), Mazatlán (n = 1050), and Los Mochis (n = 979), were interviewed (refer to Table 1 and Fig. 1). The surveyors employed a random/convenience sampling technique. This random selection process helps eliminate systematic bias and ensures that the sample's characteristics closely resemble the overall population. To achieve a broad representation encompassing various sociodemographic backgrounds, participants were randomly selected from public areas using neighborhood-dwelling maps provided by the National Institute of Geography and Statistics (INEGI). Interviewers initiated the process by randomly selecting participants aged 18 years or older. No records containing participants' names or identifiable characteristics were maintained to uphold ethical standards, protect individual identities, and minimize the potential for self-report bias. Table 1 presents the demographic characteristics of our sampled population.

**Table 1:** Features of participants (n = 3162).

Characteristic	Overall, n = 3162^1^	Consume, n = 654^1^	Do not consume, n = 2508^1^	P-value^2^
Age (years)				
18–25	829 (26%)	170 (26%)	659 (26%)	> 0.9
26–35	778 (25%)	167 (26%)	611 (24%)	
36–45	662 (21%)	140 (21%)	522 (21%)	
46–55	570 (18%)	113 (17%)	457 (18%)	
56–65	323 (10%)	64 (9.8%)	259 (10%)	
Sex				
Female	1668 (53%)	337 (52%)	1331 (53%)	0.5
Male	1494 (47%)	317 (48%)	1177 (47%)	
City				
Culiacán	1133 (36%)	227 (35%)	906 (36%)	0.2
Los Mochis	979 (31%)	222 (34%)	757 (30%)	
Mazatlán	1050 (33%)	205 (31%)	845 (34%)	
Education*				
Analphabet	167 (5.3%)	42 (6.4%)	125 (5.0%)	0.4
Elementary	1027 (32%)	208 (32%)	819 (33%)	
High school	1263 (40%)	253 (39%)	1010 (40%)	
University	674 (21%)	142 (22%)	532 (21%)	
Postgraduate	31 (1.0%)	9 (1.4%)	22 (0.9%)	

The chi-square goodness-of-fit test determined the P-value.

^1^n (%).

^2^Pearson’s Chi-squared test.

*Incomplete or complete education to this level.

### Pre-test assessment (survey phase)

Before providing scientific information on cannabis, participants' perceptions of cannabis were evaluated through a comprehensive questionnaire. The questionnaire encompassed inquiries about participants' consumption patterns and reasons for use (if applicable).

The questionnaire also aimed to characterize participants' overall attitude towards cannabis. Specifically, participants were asked to indicate whether they perceived cannabis as “bad”, “does more harm than good”, “neither bad nor good”, “does more good than harm”, or “good” (Supplementary Material).

### Intervention

Following the pre-test assessment, participants were provided with scientific information sourced from the documents “Using Evidence to Talk About Cannabis”^10^ and "State of the Evidence: cannabis use and regulation"^11^ by the International Centre for Science in Drug Policy that addressed different myths of cannabis. The surveyors underwent training to impart the information in a scholarly manner, skillfully juxtaposing prevalent cannabis claims with corresponding scientific evidence (Supplementary Material).

### Post-test assessment

After receiving the scientific information (intervention), participants' perceptions of cannabis were immediately reevaluated. This facilitated comparing pre- and post-test results to determine any shifts in participants' perceptions of cannabis.

### Analysis

Descriptive statistics were employed for data analysis to assess the demographic characteristics of the study population. The study centered on the analysis of frequency distributions and percentages. The data were categorized into distinct ranges or categories according to the variables. The age variable was subjected to data grouping based on age ranges, including but not limited to 18–25, 26–35, and so forth. The Chi-square goodness-of-fit test was utilized to assess the uniformity of consumption frequency. The Stuart-Maxwell statistical test assessed the differences in attitudes between the pre- and post-intervention groups, with stratification based on educational level. The findings were presented in bar charts, which depicted the distribution of attitudes across different groups categorized by their educational attainment. Significant differences, as determined by the Stuart-Maxwell test, were indicated with asterisks above the bars in accordance with statistical conventions. The statistical analyses and plots were conducted utilizing the R programming language and RStudio software. The significance level of 0.05 was employed to assess the rejection or acceptance of the null hypothesis.

## Results

### Demographics

The sample comprised 3162 participants, including 1668 women and 1494 men, aged 18 to 76 years (mean age = 35 years) (Table 1). In terms of educational attainment, 5.3% had no formal education, 32% completed elementary school, 40% held a high school diploma, 21% had a bachelor’s degree, and 1% had a postgraduate degree (Table 1).

### Prevalence of cannabis consumption

**Figure 2:**
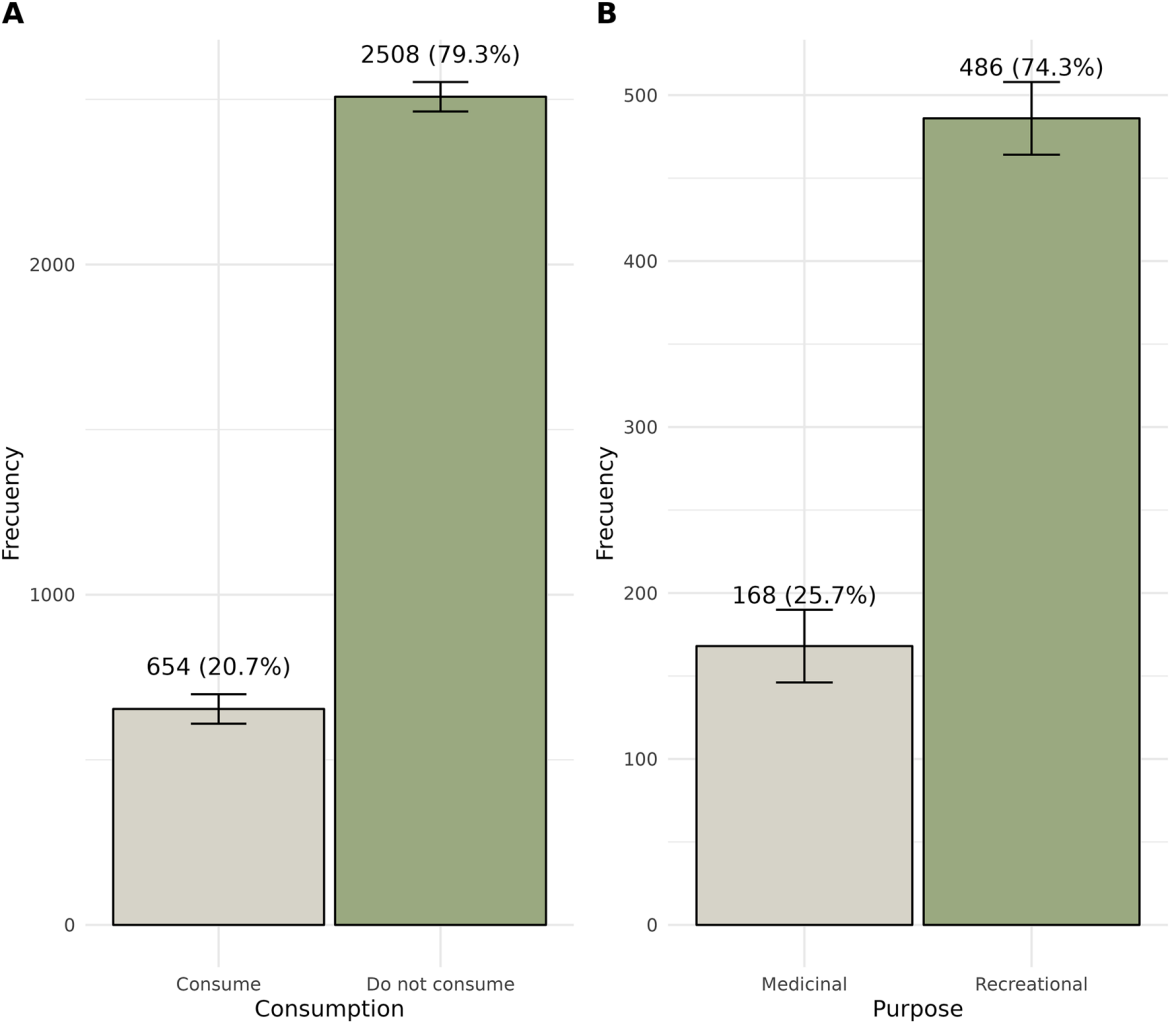
Comparative bar charts illustrating consumption patterns and purposes. The left panel (**A**) depicts the frequency of individuals who consume versus those who do not consume. The right panel (**B**) differentiates between recreational and medicinal usage. Each bar represents the frequency of respondents, with annotations indicating the exact count and corresponding percentage. Error bars denote the 95% confidence intervals for each proportion.

**Figure 3:**
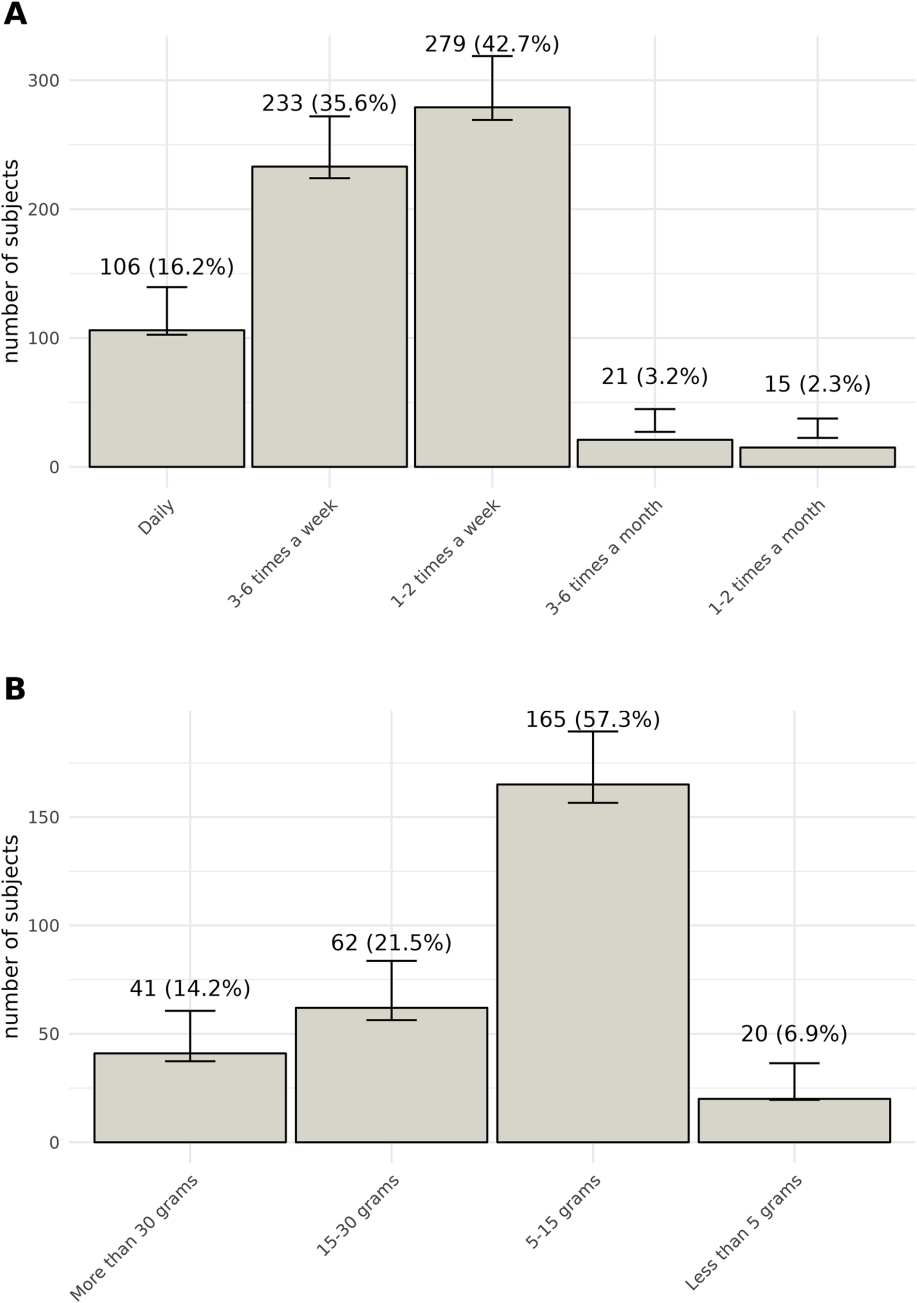
Consumption patterns of study population. This figure presents two bar charts illustrating the frequency and quantity of consumption. (**A**) shows the frequency of consumption, with categories ranging from "Daily" to "1–2 times a month". (**B**) represents the quantity consumed, with categories from "More than 30 g" to "Less than 5 g". Each bar in both charts represents the number of subjects, with annotations indicating the exact count and corresponding percentage. The error bars denote the 95% confidence intervals for each proportion.

Among the participants, only 654 (20.7%) identified themselves as active consumers of cannabis. Of these consumers, 74.3% (n = 486) reported using cannabis for recreational purposes, while 25.7% (n = 168) used it for medical reasons (Fig. 2). The majority of cannabis users reported consuming it on a weekly basis, with 42.7% (n = 279) using it 1–2 times per week, and 35.6% (n = 233) using it 3–6 times per week. Only a small percentage (16.2%) reported daily consumption (n = 106). A minority consumed cannabis on a monthly basis, with 2.3% (n = 15) using it 1–2 times per month and 3.2% (n = 21) using it 3–6 times per month (Fig. 3). Figures 2 and 3 provide an overview of the prevalence of cannabis consumption in Sinaloa.

### Attitudes towards cannabis

Our analysis delved into examining attitudes towards cannabis both before and after the intervention, with a particular focus on the influence of education and age. By stratifying the data based on these factors, we aimed to explore how different educational backgrounds and age groups may respond to the intervention and whether any significant changes in attitudes were observed.

The results of our analysis demonstrated substantial differences in attitudes towards cannabis before and after the intervention, considering taking the participants' educational levels and age categories. These differences were statistically significant, indicating that the intervention had a noticeable impact on shaping attitudes towards cannabis among different population segments.

**Figure 4:**
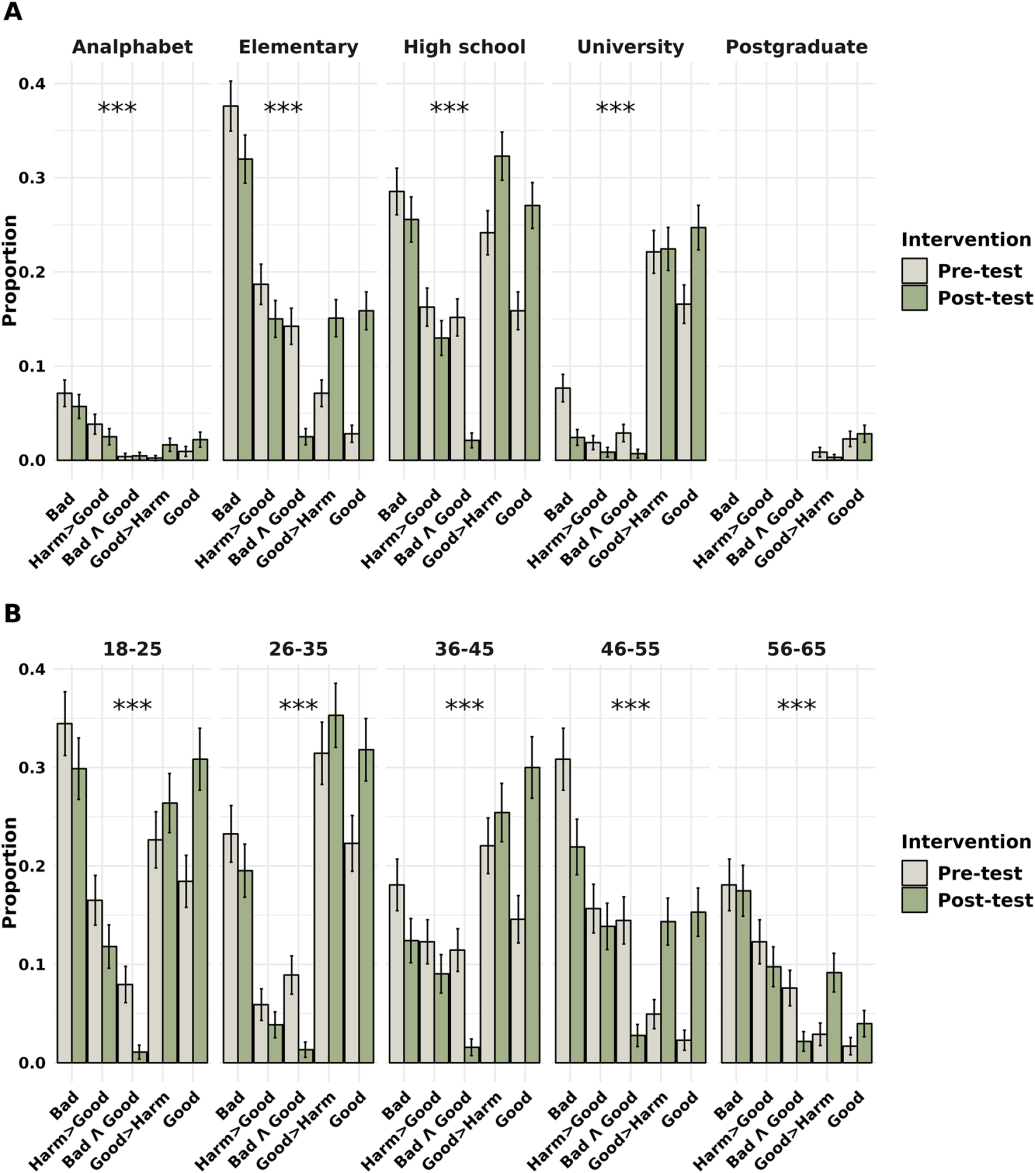
Proportions of attitudes towards educational level (**A**), age group (**B**), and intervention. The asterisks (*) above the bars indicate the statistical significance of the differences between the Pre-intervention and Post-intervention groups based on the Stuart-Maxwell test, with *p < 0.05, **p < 0.01, and ***p < 0.001. The error bars denote the 95% confidence intervals for each proportion.

When considering the educational background, we observed that individuals with varying levels of education displayed distinct changes in their attitudes toward cannabis following the intervention. In Panel A of Fig. 4, we observed statistically significant shifts in attitudes across all educational groups, as denoted by p-values < 0.05 (indicated by asterisks). Notably, the intervention had distinct effects on individuals from different educational backgrounds, with non-uniform changes in attitudes observed. Specifically, the "Analphabet" and "Elementary" education groups experienced a significant transition toward more positive attitudes following the intervention. In the "High School," "University," and "Postgraduate" groups, there was a significant increase in more neutral attitudes towards cannabis.

Regarding age categories, our analysis unveiled significant changes in attitudes towards cannabis associated with different age groups. In Panel B of Fig. 4, we examined the variations in attitudes across different age categories. Similar to the educational stratification, age groups exhibited significant changes in attitudes towards cannabis following the intervention. The younger age groups demonstrated a significant shift towards more positive attitudes, whereas the older age groups tended to adopt a more neutral stance after the intervention. The Stuart-Maxwell test confirmed the statistical significance of these changes, as indicated by the p-values for each age group.

In addition, among consumers of cannabis, the intervention yielded a noteworthy transformation in attitudes. Prior to receiving scientific information, a majority perceived cannabis as "bad" (328 individuals), whereas after the intervention, this number decreased to 260 individuals. Moreover, the number of individuals categorizing cannabis as "good" increased from 37 to 131 (Table 2). Similar trends were observed among non-consumers of cannabis. Prior to the intervention, the perception of cannabis as "bad" was prominent (734 individuals), which decreased to 597 after the intervention. The number of individuals who viewed cannabis as "good" increased from 452 to 829 (Table 2).

**Table 2:** Consumers vs non-consumers (n = 3162).

Attitude toward cannabis	Consume, n = 654^1^	Do not consume, n = 2508^1^	P-value^2^
Response pre intervention			
Good	37 (5.7%)	452 (18%)	< 0.001
Bad	328 (50%)	734 (29%)	
Good > Harm	42 (6.4%)	617 (25%)	
Harm > Good	150 (23%)	366 (15%)	
BadΛGood	97 (15%)	339 (14%)	
Response post intervention			
Good	131 (20%)	829 (33%)	< 0.001
Bad	260 (40%)	597 (24%)	
Good > Harm	112 (17%)	772 (31%)	
Harm > Good	134 (20%)	261 (10%)	
BadΛGood	17 (2.6%)	49 (2.0%)	

The chi-square goodness-of-fit test determined the P-value.

^1^n (%).

^2^Pearson’s Chi-squared test.

## Discussion

The prevalence of cannabis consumption among the participants in this study presents noteworthy insights into the patterns and purposes of use within the state of Sinaloa, Mexico. Out of the total participants, a relatively small but still significant proportion of 20.7% identified themselves as active consumers of cannabis. Such figures indicate that cannabis remains a relevant and relevantly used substance within the sampled population.

Further examination of these active consumers revealed intriguing distinctions in their motivations for using cannabis. The majority, comprising 74.3% of users, reported employing cannabis for recreational purposes. This finding aligns with the notion that recreational use constitutes a primary driver of cannabis consumption in this region, implying that it may be a preferred choice for relaxation, leisure, or social interaction. Conversely, 25.7% of active consumers cited using cannabis for medical reasons. This significant segment of individuals highlights the growing recognition and acceptance of cannabis as a potential therapeutic option, with users potentially seeking relief from various medical conditions. The presence of a substantial proportion of medical users signifies a shifting perspective on cannabis, as more individuals explore its potential medicinal benefits.

Regarding the frequency of consumption, the data reveals compelling patterns. A notable 42.7% of users reported using cannabis 1–2 times per week, signifying a regular but moderate consumption pattern among a significant portion of the active consumers. Additionally, 35.6% of users indicated consuming cannabis 3–6 times per week, suggesting a slightly higher frequency of use among this subset. Notably, a minority, comprising 16.2% of active consumers, reported daily consumption of cannabis. This finding implies that while some individuals engage in more frequent use, the majority of cannabis users do not resort to daily consumption, potentially indicating a more moderate and measured approach to its use. Furthermore, the data highlights a smaller subset of users who consume cannabis on a monthly basis. Merely 2.3% of users reported using it 1–2 times per month, and 3.2% indicated consumption 3–6 times per month. This infrequent use pattern among a minority of users suggests that for most cannabis consumers in Sinaloa, cannabis use is more consistently integrated into their routines.

Supplementing the prevalence outcomes, it becomes imperative to delve into the wider societal perspective of cannabis within Sinaloa, Mexico. These results assume particular significance in light of the intervention's implications. The prevailing attitudes and beliefs surrounding cannabis use could significantly influence public policies and social norms^12^. This comprehensive evaluation of prevalence and perception not only unveils a rounded understanding of cannabis's position within Sinaloa's societal context but also underscores the pivotal implications of the intervention's outcomes.

The results of our study reveal the intricate relationship between educational background and the impact of our intervention on attitudes towards cannabis. A detailed analysis of educational groups, as depicted in Panel A of Fig. 4, revealed conspicuous alterations in attitudes following the intervention. Notably, the intervention yielded nuanced effects across distinct educational strata, characterized by non-uniform changes in attitudes. Particularly intriguing was the discernible shift towards more positive attitudes in the "Analphabet" and "Elementary" education groups subsequent to the intervention. Similarly, among the "High School," "University," and "Postgraduate" education groups, a statistically significant trend towards more neutral attitudes was observed.

Turning our focus to age categories, our investigation brought to light substantial modifications in attitudes towards cannabis associated with different age cohorts. Illustrated in Panel B of Fig. 4, the assessment of attitude variations across diverse age groups mirrored the patterns observed in educational stratification. Much akin to education's influence, age emerged as another factor influencing the efficacy of the intervention. Specifically, the younger age groups demonstrated a significant shift towards more positive attitudes post-intervention. Conversely, the older age groups exhibited a propensity towards adopting a more neutral perspective after the intervention. These findings underscore the role of age in mediating the impact of the intervention on attitudes towards cannabis.

Disseminating factual information can potentially modify public opinion by providing individuals with accurate and evidence-based knowledge on a specific topic. When presented with accurate information, supported by research and evidence, people have the opportunity to adjust their opinions and beliefs accordingly. Additionally, factual information can help challenge and correct misinformation that may have contributed to inaccurate or negative public opinions. By introducing new information that individuals may not have previously considered or been aware of, public opinion can be influenced and changed^12^.

Furthermore, the use of factual information can enhance credibility and trust with the public, leading to a positive shift in public opinion. It can also raise awareness about a particular issue, generating increased public concern and support for taking action. Providing scientific information allows individuals to develop a better understanding of potential benefits and risks^13^. Accurate scientific information empowers individuals to make informed decisions, reducing associated risks and promoting responsible consumption. Scientific information also serves to dispel myths and misconceptions about controversial topics, like cannabis, reducing stigma and fostering a more informed and compassionate society^14^.

Our study revealed that age significantly impacts attitudes towards cannabis perception. Different generations may hold distinct attitudes towards cannabis due to variations in upbringing, cultural values, and exposure to cannabis. For example, older generations may have negative perceptions of cannabis due to its association with illegal drug use, while younger generations may be more accepting due to changing attitudes towards legalization. Aging individuals may become more concerned about their health and the potential negative effects of cannabis use, which can lead to more negative attitudes toward cannabis perception^15^. Furthermore, social circles that include regular cannabis users may influence attitudes towards cannabis. Younger individuals, exposed to cannabis use among peers, may hold more positive attitudes towards its use.

Moreover, the educational level also significantly influences attitudes toward cannabis perception. Individuals with higher education levels tend to possess more knowledge and awareness about different topics, facilitating proficient information processing capabilities. This may result in more nuanced and informed attitudes. Cultural values associated with higher education can impact attitudes towards cannabis, with more accepting attitudes among individuals with liberal or progressive cultural values^16^. Conversely, lower levels of education may be associated with stigmatizing attitudes or susceptibility to misinformation. Our findings demonstrate the substantial impact of the intervention on attitudes toward cannabis use, with age and education playing significant roles in its effectiveness. The intervention fostered more positive or more neutral attitudes, reducing stigma and promoting an informed perspective on cannabis use.

Cannabis is a multifaceted substance, and its effects on individuals can vary significantly depending on factors such as the mode of consumption, dosage, frequency of use, and individual physiology. Personal experiences with cannabis and the cultural and societal norms surrounding it can shape people's perception of the substance. Interestingly, we found that among those who consume cannabis, the intervention brought about a significant shift in perspectives. Prior to being exposed to scientific information and despite being active consumers of cannabis, a substantial majority held a negative view of cannabis, with 328 individuals categorizing it as "bad." However, post-intervention, this count decreased to 260. Correspondingly, the group recognizing cannabis as "good" increased from 37 to 131. This pattern suggests that the provided scientific information contributed to a more favorable view of cannabis among consumers, potentially altering perceptions of its impact.

Comparable patterns were discerned among individuals who do not consume cannabis. Preceding the intervention, the prevalent perception of cannabis as "bad" was evident among 734 individuals, but this number diminished to 597 following the intervention. The cohort endorsing a "good" characterization of cannabis expanded from 452 to 829. These findings suggest that the intervention also influenced non-consumers' attitudes towards cannabis, leading to a more positive disposition.

The observed changes in the categories "cannabis does more good than harm" and "cannabis does more harm than good" further accentuate the impact of the intervention. Among consumers, a shift from perceiving cannabis as causing “more harm than good” (150 individuals to 134 individuals) to believing it does “more good than harm” (42 individuals to 112 individuals) was evident. Similarly, among non-consumers, the shift from cannabis does “more harm than good" (366 individuals to 261 individuals) to cannabis does “more good than harm" (617 individuals to 772 individuals) was pronounced. These transitions underscore the role of scientific information in altering the perceived balance of benefits and harms associated with cannabis.

However, it is noteworthy that, overall, less participants categorized cannabis as "neither good nor bad" after the intervention (Table 2). This could reflect a tendency to lean towards positive or negative categorizations, suggesting that participants might associate strong valuations with cannabis despite the information provided. Further investigation into the underlying factors influencing these categorizations could provide insights into the complexities of cannabis perception.

Nevertheless, while the results of this study shed light on the prevalence and attitudes towards cannabis consumption in Sinaloa, Mexico, it is essential to consider potential biases that may have influenced these findings. The study utilized a large convenience sample of participants from the main cities in Sinaloa. However, certain populations or subgroups, such as individuals with limited access to public areas or those who may be more hesitant to participate, might have been underrepresented. This sampling bias could impact the findings' generalizability to the entire Sinaloa population. In addition, the questionnaire used to assess cannabis consumption and attitudes relied on participants' recall of their past behaviors and experiences. This reliance on memory could introduce recall bias, leading to inaccuracies in reporting the frequency or quantity of cannabis use and potentially skewing the results. Furthermore, the method of verbal interviewing with participants could have introduced social desirability bias, where participants may have been inclined to respond in a manner they perceived as socially acceptable or desirable. This bias could potentially influence their reporting of cannabis consumption and attitudes, leading to an underreporting of illicit drug use or an over-reporting of more socially accepted attitudes. Moreover, participants who willingly agreed to participate in this study might have different attitudes or experiences related to cannabis compared to those who declined to participate. This self-selection bias could lead to a non-representative sample that may not accurately reflect the broader population's attitudes and behaviors.

In addition, our reliance solely on information from the International Centre for Science in Drug Policy^10,11^, while providing valuable scientific insights about cannabis, introduces some limitations. Relying on a single source for information may inadvertently lead to potential bias or lack of comprehensive coverage of all available research on the topic. Different organizations and institutions may have varying perspectives, methodologies, and interpretations of scientific data, and excluding other reputable sources might limit the diversity of evidence considered. Additionally, by using information solely from one organization, the study might not capture the most current or cutting-edge research in the rapidly evolving field of cannabis science. To enhance the study's robustness and validity, future research could incorporate a more extensive range of credible sources, ensuring a more well-rounded and comprehensive understanding of cannabis-related scientific evidence.

Furthermore, this study lacks specific qualitative data that could provide more in-depth insights into these changing attitudes. Qualitative investigations could potentially uncover underlying factors that contribute to the shifts observed in different educational and age strata. This could encompass aspects such as exposure to diverse information sources, social influences, and personal experiences that may influence the varying attitudes within each group. Such an exploration could provide valuable qualitative context to complement our quantitative findings and offer a more comprehensive understanding of the dynamics at play in shaping attitudes towards cannabis among distinct backgrounds.

Finally, the study was conducted in Sinaloa, which has its unique cultural and social context. The prevailing societal norms, values, and perceptions about cannabis in Sinaloa may differ from other regions in Mexico or globally. Thus, the findings might not be generalizable to areas with different cultural backgrounds or drug policies. Interestingly, despite the vast expanse of Mexico, comprising diverse communities with unique perspectives, the findings observed in Sinaloa regarding attitudes toward cannabis hold substantial similarities with those across the rest of the country. The National Telephone Survey conducted in 2020 by the Center for Social Studies and Public Opinion of the Mexican Congress of the Union, Chamber of Deputies, yielded results that align with the findings of this study (50.5% disapproves cannabis, 46.3% approves cannabis, 3.2% did not respond)^17^. Despite regional variations in cultural norms and societal influences, overarching factors, such as the prohibitionist history and pervasive drug trade, have contributed to a pervasive narrative surrounding cannabis perception that resonates throughout Mexico.

The effectiveness of providing scientific information in fostering more positive or more neutral attitudes towards cannabis has demonstrated potential for reducing stigma and enhancing public understanding of the subject. This phenomenon, observed in Sinaloa, is reflective of a broader societal inclination to embrace well-founded evidence and dispel unfounded myths and misconceptions surrounding cannabis.

It is worth mentioning that a comparative analysis of the potential effectiveness of distinct interventions or strategies such as educational workshops, multimedia campaigns, or focus groups, could offer a unique opportunity to unveil the diverse impacts these interventions might exert on attitudes. Comparing these diverse interventions could provide valuable insights into which approach holds the highest efficacy in effecting attitude change towards cannabis. By considering the multifaceted nature of attitude formation and transformation, a comprehensive evaluation can guide the selection of interventions that are best suited to target specific audiences and achieve lasting shifts in perceptions.

In light of the evolving cannabis regulations in Mexico, engaging knowledgeable individuals in significant policy-making decisions is crucial to ensure effective and credible policies. Our findings substantiate the notion that the provision of accurate and evidence-based information can effectively elicit positive modifications in perceptions related to cannabis.

Given Sinaloa's history as a prominent hub for drug trafficking in the Americas, tailored state policies are necessary to address the violence and inequalities associated with the drug trade. The grave societal consequences of decades of prohibitionist strategies and state corruption have shaped public discourse in several ways. There is a growing recognition of the failure of the traditional prohibitionist approach, with its emphasis on punitive measures and its failure to address the root causes of drug abuse and trafficking. This has sparked discussions on alternative approaches, such as harm reduction, decriminalization, and drug policy reform, prioritizing public health and social welfare^5,6^. In addition, civilian casualties and the collateral damage from drug-related violence have increased demand for more compassionate and evidence-based drug policies. Calls for addressing drug abuse as a public health rather than a criminal have gained traction, as society recognizes the need for a more balanced and pragmatic approach to drug control.

A well-informed society regarding cannabis could catalyze the designing of effective policies, fostering transparency, and upholding governmental accountability. It also might engender a rich tapestry of public opinion, reflecting diverse perspectives and values. Providing accurate information empowers citizens, enabling them to make informed choices, offer constructive insights, and hold elected representatives answerable for their policy decisions. This active engagement constitutes an indispensable foundation for crafting policies that resonate with the needs of the entire community while mitigating unintended repercussions and ensuring the successful execution of transformative initiatives.

### Supplementary Information

Supplementary Information.

## Data Availability

The data generated and analyzed during the course of this study are available upon request. Access to the data can be granted upon submission of a formal request to the corresponding author of the study. Requests for data access will be reviewed and evaluated to ensure that they align with the ethical considerations and privacy concerns surrounding the data. To request access to the data, interested parties should contact Alberto K. De la Herrán-Arita at alberto.kousuke@uas.edu.mx. The data will be made available in compliance with applicable laws, regulations, and institutional policies governing data sharing and protection.
