# Supplementary material for: Scientific facts improve cannabis perception and public opinion: results from Sinaloa, México
Source: Sci Rep. 2023 Oct 12;13:17318. doi: 10.1038/s41598-023-44185-5 (PMC10570375; doi:10.1038/s41598-023-44185-5)
Supplement: Supplementary file 1 — Supplementary Information. [file 41598_2023_44185_MOESM1_ESM.docx]

**QUESTIONNAIRE**

**Sex: __________ Age: __________ City: __________ Sheet number: __________ Date: __________**

**1. Education level**

**Analphabet ___ Elementary___ Highschool___ University___ Postgraduate___**

**2. Which drug did you try first?**

**Alcohol ___ Tobacco___ Cannabis___ Cocaine___ Opioids___ Hallucinogens___ Solvents___ None___**

**3. How much cannabis do you consume per month?**

**I do not ___ Less than 5 grams___ 5-15 grams___ 15-30 grams___ More than 30 grams___**

***If the respondent does not consume cannabis, omit questions number 4 and number 5**

**4. How often do you consume cannabis?**

**1-2 times/month ___ 3-6 times/month ___ 1-2 times/week___ 3-6 times/week___ Daily___**

**5. For what reasons do you consume cannabis?**

**Recreation ___ Medical ___**

**6. Do you think cannabis is?**

**Bad ___ Does more harm than good ___ Nor bad nor good___ Does more good than harm___ Good__**

**- APPLY INTERVENTION (INFORMATION) -**

**7. Do you think cannabis is?**

**Bad ___ Does more harm than good ___ Nor bad nor good___ Does more good than harm___ Good__**

***The questionnaire encompassed supplementary inquiries. The present set of queries pertains exclusively to the focus of this study.**

Remember the crucial importance of conveying the information as literally and objectively as possible. Your role is to be a conduit for the respondents' input without any interpretation or personal bias. When communicating information or recording responses, strive for clarity and precision. It is of utmost importance to explicitly state that the information you present is firmly rooted in scientific data. Emphasize this fact to respondents, conveying that their responses will contribute to a rigorous and evidence-based analysis.

When conducting surveys, please approach each task with an objective mindset, free from personal biases or preconceived notions. Prioritize neutrality throughout the process, refraining from any actions or statements that could potentially influence respondents' answers.

Thank you for your dedication to upholding these high standards.

**INTERVENTION**

**State of the evidence: cannabis use and regulation / Using evidence to talk about cannabis**

**International Centre for Science in Drug Policy (ICSDP)**

| **Claim** | **Evidence** |
| --- | --- |
| *Common claims on cannabis use* | |
| Cannabis is as addictive as heroin. | A lifetime of cannabis use carries a low risk of dependance (9%), while the risk of cannabis dependence is very low among those who report using it for one year (2%) or even 10 years (5.9%). This is much lower than the estimated lifetime risk of dependence to heroin (23.1%). |
| Marijuana acts as a gateway drug. | Evidence to date does not support the claim that cannabis use causes subsequent use of “harder” drugs. |
| Cannabis use can cause potentially lethal damage to the heart and arteries. | There is little evidence to suggest that cannabis use can cause lethal damage to the heart, nor is there clear evidence of an association between cannabis use and cancer. |
| Cannabis use lowers IQ by up to 8 points. | There is little scientific evidence suggesting that cannabis use is associated with declines in IQ. |
| Cannabis use impairs cognitive function. | While the evidence suggests that cannabis use (particularly among youth) likely impacts cognitive function, the evidence to date remains inconsistent regarding the severity, persistence, and reversibility of these cognitive effects. |
| Cannabis is a drug that can result in serious, long-term consequences, like schizophrenia. | While scientific evidence supports an association between cannabis use and schizophrenia, a causal relationship has not been established. |
| *Common claims on cannabis regulation* | |
| Legalization/regulation increases the availability of cannabis. | Evidence suggests that the supply of illegal cannabis has increased under a prohibition model, and that availability has remained high among youth. Evidence does not suggest that cannabis availability among youth has increased under regulatory systems. |
| If marijuana was legalized, the increase in users would be both large and rapid. | Evidence suggests that the policy environment (specifically legal status and enforcement policy) has at most a marginal impact on the prevalence of drug use, thereby suggesting that regulating cannabis markets will not inevitably cause higher levels of cannabis use. |
| Regulation will not reduce drug crime. | Given that the prohibition of cannabis has not been shown to reduce illegal supply, it is likely that cannabis regulation is more effective at minimizing criminal markets for cannabis, despite the fact that criminal markets will continue to represent a proportion of the total market. |
| We are going to have a lot more people stoned on the highway and there will be consequences. | While experimental studies suggest that cannabis intoxication reduces motor skills and likely increases the risk of motor vehicle collisions, there is not sufficient data to suggest that cannabis regulation would increase impaired driving, and thereby traffic fatalities. |
| Regulation promotes drug tourism. | While evidence suggests that, depending on the use of regulatory controls and geographic setting, regulation may in some cases lead to an increase in drug tourism, the data do not suggest that this is an inevitable consequence of regulation. |
| Regulation leads to a “Big Marijuana” scenario. | Available evidence regarding “Big Marijuana” is currently lacking, though regulatory controls can be introduced within regulatory systems to reduce the potential of profit maximization by cannabis retailers. |
